# Social health insurance contributes to universal coverage in South Africa, but generates inequities: survey among members of a government employee insurance scheme

**DOI:** 10.1186/s12939-017-0710-z

**Published:** 2018-01-04

**Authors:** Jane Goudge, Olufunke A. Alaba, Veloshnee Govender, Bronwyn Harris, Nonhlanhla Nxumalo, Matthew F. Chersich

**Affiliations:** 1 Centre for Health Policy/MRC Health Policy Research Group, School of Public Health, Faculty of Health Sciences, University of the Witwatersrand, Johannesburg, South Africa; 2 Health Economics Unit, School of Public Health and Family Medicine, University of Cape Town, Cape Town, South Africa; 3 Wits Reproductive Health and HIV Institute, Faculty of Health Sciences, University of Witwatersrand, Johannesburg, South Africa

**Keywords:** Access, Universal health coverage, Utilization, Social health insurance, South Africa, Government employees

## Abstract

**Background:**

Many low- and middle-income countries are reforming their health financing mechanisms as part of broader strategies to achieve universal health coverage (UHC). Voluntary social health insurance, despite evidence of resulting inequities, is attractive to policy makers as it generates additional funds for health, and provides access to a greater range of benefits for the formally employed. The South African government introduced a voluntary health insurance scheme (GEMS) for government employees in 2005 with the aim of improving access to care and extending health coverage. In this paper we ask whether the new scheme has assisted in efforts to move towards UHC.

**Methods:**

Using a cross-sectional survey across four of South Africa’s nine provinces, we interviewed 1329 government employees, from the education and health sectors. Data were collected on socio-demographics, insurance coverage, health status and utilisation of health care. Multivariate logistic regression was used to determine if service utilisation was associated with insurance status.

**Results:**

A quarter of respondents remained uninsured, even higher among 20–29 year olds (46%) and lower-skilled employees (58%). In multivariate analysis, the odds of an outpatient visit and hospital admission for the uninsured was 0.3 fold that of the insured. Cross-subsidisation within the scheme has provided lower-paid civil servants with improved access to outpatient care at private facilities and chronic medication, where their outpatient (0.54 visits/month) and inpatient utilisation (10.1%/year) approximates that of the overall population (29.4/month and 12.2% respectively). The scheme, however, generated inequities in utilisation among its members due to its differential benefit packages, with, for example, those with the most benefits having 1.0 outpatient visits/month compared to 0.6/month with lowest benefits.

**Conclusions:**

By introducing the scheme, the government chose to prioritise access to private sector care for government employees, over improving the availability and quality of public sector services available to all. Government has recently regained its focus on achieving UHC through the public system, but is unlikely to discontinue GEMS, which is now firmly established. The inequities generated by the scheme have thus been institutionalised within the country’s financing system, and warrant attention. Raising scheme uptake and reducing differentials between benefit packages will ameliorate inequities within civil servants, but not across the country as a whole.

## Background

Globally, calls to provide inclusive, equitable and quality health care for all at affordable cost – universal health care (UHC) – have gained momentum [1–4]. Many low- and middle-income countries (LIMCs) are experimenting with different forms of health financing reforms as part of broader strategies to achieve UHC. These include increased funding from taxation, national mandatory schemes or voluntary social health insurance schemes. (See [5] for a review of financing mechanisms in selected LMICs). Recent LMIC experience suggests it is possible to achieve UHC with various, often employment-based, insurance schemes, when complemented by sufficient tax funding to subsidise membership for the unemployed and those unable to afford the premium. For example, Thailand, often described as an example of a successful transition to UHC, followed this path [6]. However, the result is a patchwork of schemes with different benefit packages. These often entrench inequities by providing differential access to services for different groups of the population, thereby hindering achievement of ‘equitable access to quality care for all’ [7]. Thus a key question is, if faced with limited tax funding, should Ministries of Health consider establishing employment-based schemes (with cross-subsidisation that is internal to the scheme, such as between the higher- and the lower-paid workers) as a medium-term solution, which provides greater insurance coverage for some? Or, should countries avoid establishing employment-based insurance schemes and use available tax funding to strengthen public sector services, which then can serve as an equitable foundation for their health care financing system, even if this means a smaller package of care for everybody [7–10]?

In 2005, the South Africa government introduced the Government Employees Medical Scheme (GEMS), a health insurance scheme for government employees, which successfully raised the insurance coverage among civil servants [11, 12]. More recently, other financing reforms have been mooted (a centralised pooling of general tax revenue and value-added tax, a purchasing fund and benefit specification). Yet, despite a government White Paper on National Health Insurance in 2015, there has been little progress towards finalisation of these reforms [13], and the publicly-subsidized GEMS continues alongside a number of fragmentary private insurance schemes.

In this paper we ask whether the GEMS scheme has further institutionalised inequities in access to health care. Firstly, we examine whether insurance status and socio-economic status (measured as skill levels, which correlates strongly with income) influenced access to care. This involved assessing whether the take up of insurance varied with health status, and comparing the health service utilisation of members of the GEMS scheme with their uninsured colleagues. Particular attention is paid to examining whether membership of the subsidised lower-cost insurance packages have enabled similar access (thereby improving horizontal equity^1^) for lower paid compared-to higher-paid members. Secondly, we investigate whether the design of the scheme, based around differential benefit packages, has contributed to inequities in utilisation of services. These findings will assist other countries trying to balance the trade-offs between increasing the number of people with insurance in the country, and therefore improving access to care for some, against the inequities that ensue. A brief summary of the health financing in South Africa and the GEMS structure is provided.

### Background to health financing in South Africa

Inequitable access to health care is a key problem facing the South African health system. Tax-funded public services are utilized by the majority (84%) of the population [14]. This majority (who do not have insurance) often pay out-of-pocket to use private sector general practitioners (GPs) and pharmacies [15], due to the perceived poor quality of care in the public sector. The remainder of the population (16%) are privately insured and use a well-developed private delivery system [16].

The National Department of Health began exploring the feasibility of various financing reforms after the transition to democracy in 1994. (See [17] for detailed policy initiatives and proposal timelines). GEMS, a voluntary and subsidized government employee medical scheme was established in 2005 to provide “all public service employees with *equitable* access to affordable and comprehensive health care benefits” [18] (our italics). GEMS has five benefit packages ranging from low-cost options that are fully subsidised for lower income employees, to high-cost alternatives. The Sapphire and Beryl packages offer members outpatient benefits through a limited network of private providers (general practitioners, dentists and optometrists).^2^ Sapphire and Beryl, however, differ with respect to hospital benefits; Sapphire members are required to use state hospitals, while Beryl offers access to a limited private hospital network. Sapphire is fully subsidised for the lowest paid employees and there is consequently no financial reason for members of this group to remain uninsured, although not all have taken up membership [19]. The remaining three options (Ruby, Emerald and Onyx) are differentiated by the comprehensiveness of their benefit packages for outpatient services, but all allow access to any private hospital for inpatient care [17].^3^

## Methods

### Sampling and data collection

Across four of South Africa’s nine provinces, 1329 government employees were interviewed in 2008–9^4^ (full details of study methods are detailed elsewhere) [12, 20]. Health and education, two of the largest public sectors, were selected for the survey. To assess variation in utilisation related to geographical access, two provinces (Gauteng and Western Cape) were chosen on the basis of being urban, having a greater distribution of private providers and relatively well-resourced public health care facilities, and two (KwaZulu-Natal and North West Provinces) were selected for being predominantly rural, with few private facilities and less-resourced public facilities. The minimum sample size per province was 245 and this was inflated to 309 to allow for possible incomplete questionnaires.

Multi-stage random sampling was used. First, the number of health and education employees to be sampled in each salary category was determined by their relative proportion in each province. Second, districts in each province were selected with a probability proportionate to number of employees, following which 15 schools and 4 hospitals within each of the selected districts were randomly selected. Finally, within the selected schools and hospitals, a sampling frame was constructed of all employees, stratified by salary category, to allow specific quotas of interviews to be conducted across the different salary categories. Study procedures received ethics clearance from University the Witwatersrand (Human Research Ethics Committee (Medical) Certificate number M080103), as well as permissions from the relevant Provincial Department of Health bodies. All respondents provided signed informed consent.

### Study variables and data analysis

Information was collected on health insurance membership, and classified as uninsured, privately insured (pre-paid schemes other than GEMS) and GEMS membership. GEMS membership was further disaggregated by type of benefit packages chosen, as described above. In analysis, three packages (Sapphire, Beryl and Ruby) were grouped together as ‘lower-cost’; with Emerald categorised as ‘mid-range’ and Onyx, ‘high-cost’. Participant’s need for care was measured by self-rated health status, assessed as excellent, good, average, poor and very poor (the last two categories were combined). Access to care was measured by utilisation of outpatient services (mean visits/person in past month) and inpatient services (admitted to either a hospital or clinic for one or more nights in the past year). We used skill level of respondents as a measure of socio-economic status, as this determines salary levels, which vary considerably across bands: lower skilled (USD5,688–7109); skilled (USD7,317–11,913); highly skilled production (USD12,577–22,588); highly-skilled supervision (USD23,232–63,034); and management (USD72,057–142,400). We combined the last two classes due to small sample size.

The data were double-entered by an independent survey company, cross-checked by the research team and then analysed using Stata® 12 (Stata Corporation, College Station, TX, United States). Comparisons of the means of continuous variables were performed using the ANOVA test. The frequency distributions were calculated and the chi-square test was used to assess associations between groups. Sample weights were not applied as the sampling strategy was designed such that each health and education employee in the four provinces that participated had an equal probability of being selected for the survey [21].

To identify inequities, we assessed the degree to which need and access align across different socio-economic groups. This involved several steps. Firstly, determining whether insurance status was associated with socio-economic status (Table 1), need (Table 2) and service utilisation (Tables 3 & 4). We report these associations among the total study sample and in the lower-skilled workers, as well as examine variations in access across the different GEMS packages. Lastly, using multivariate logistic regression modelling, we examined whether service utilisation was associated with insurance status, after adjusting for need for services, and factors such as age, gender, race, province socio-economic status and sector (Tables 5 & 6). Four logistic models were constructed for the outcome service utilization, which was operationalised as: whether an employee used any outpatient (i) or inpatient service (ii), or any private (iii) or public outpatient service (iv). Variables associated with the outcome in univariate analysis (*P* < 0.1) were included in the initial model and retained if their removal markedly altered the model’s fit.

**Table 1:** Distribution of health insurance status across by socio-demographics and salary grade

Variable (n)		Uninsured (342)	Privately Insured (574)	GEMS (408)	GEMS OPTIONS			Total sample (1330)*
					Sapphire/ Beryl/Ruby ‘low-cost’ (67)	Emerald ‘mid-cost’ (305)	Onyx ‘high-cost’ (36)	
		Row %						Col %
Age	20–29 (141)	46.1	22.0	31.9	13.3	84.4	2.2	10.6
	30–39 (402)	25.6	40.8	33.6	16.4	78.4	5.2	30.3
	40–49 (468)	21.4	49.2	29.5	17.5	75.9	6.6	35.3
	50–69 (314)	22.9	46.8	30.3*	16.5	62.6	20.9*	23.7
Gender	Female (778)	21.2	46.0	32.8	14.2	78.4	7.5	58.7
	Male (548)	32.3	38.9	28.8	20.3	68.6	11.1**	41.3
Race	Black (858)	28.9	39.9	31.2	16.7	80.3	3.0	65.0
	Indian (77)	13.0	52.0	35.1	21.3	69.3	9.3	5.8
	Mixed ancestry (253)	29.3	40.3	30.4	11.1	70.4	18.5	19.2
	White (132)	6.1	63.6	30.3*	10.0	50.0	40.0*	10.0
Marital status	Married/cohabiting (806)	22.1	48.3	29.5	16.7	73.0	10.3	60.6
	Divorce/widow/separate (149)	22.8	40.9	36.2	24.1	59.3	16.7	11.2
	Single (375)	34.4	33.1	32.5*	12.4	85.1	2.5*	28.2
Education	None-primary complete (168)	51.2	25.0	23.8	44.7	55.3	0	12.6
	Incomplete secondary (102)	33.3	34.3	32.4	21.2	78.8	0	7.7
	Completed secondary (184)	37.0	23.4	39.7	19.4	72.2	8.3	13.8
	Tertiary (876)	17.6	51.8	30.6*	10.9	77.7	11.3*	65.9
Sector	Health (486)	35.0	34.4	30.7	17.8	76.7	5.5	36.5
	Education (844)	20.4	48.2	31.4*	15.7	73.7	10.7	63.5
Province	Western Cape (343)	27.1	42.6	30.0	20.8	63.4	15.8	25.8
	KwaZulu Natal (310)	27.4	41.9	30.7	13.7	83.2	3.2	23.3
	North West (331)	18.1	53.2	28.7	18.1	76.6	10.2	24.9
	Gauteng (345)	29.9	35.1	35.1*	13.6	76.3	10.2*	26.0
Household income	Median per month Interquartile range	400 267–667	667 400–1333	547* 293–933	400 213–667	647 353–933	933* 400–1667	533 333–1067
Salary grade	Lower-skilled (168)	57.7	19.6	22.6	44.4	55.6	0	12.6
	Skilled (246)	39.4	21.1	39.4	21.9	75.0	3.1	18.5
	Highly skilled (709)	18.8	50.5	30.8	12.0	80.6	7.4	53.4
	Management (206)	7.3	63.1	29.6*	6.7	65.0	28.3*	15.5
Total		25.8	43.4	30.8	16.4	74.8	8.8	100

*P < 0.05. **P = 0.05–0.10. Sum of totals may be less than the total sample (1330) due to missing data

**Table 2:** Self-reported health status in the whole study sample and in lower skilled workers, by insurance status

Variable		Uninsured	Privately Insured	GEMS	GEMS OPTIONS			
					Sapphire/ Beryl/Ruby ‘low-cost’	Emerald ‘mid-cost’	Onyx ‘high-cost’	Total sample
Total sample		*N* = 342	*N* = 574	*N* = 408	*N* = 67	*N* = 305	*N* = 36	*N* = 1330
Health status	Excellent (320)	27.0	20.9	26.1	32.8	23.9	33.3	24.1
	Good (633)	44.9	49.1	47.8	34.3	49.8	52.8	47.6
	Average (342)	25.5	28.2	22.5	31.3	22.6	8.3	25.7
	Poor or very poor (34)	2.6	1.7	3.6**	1.5	3.6	5.6**	2.6
Percent had illness in last month (380)		17.3	33.1	32.0*	28.4	31.9	40.0	28.7
Lower skilled workers		*N* = 97	*N* = 33	N = 36	*N* = 16	*N* = 20	*N* = 0	*N* = 168
Health status	Excellent (23)	15.6	9.1	13.2	25.0	5.0	–	13.8
	Good (73)	44.8	45.5	39.5	43.8	35.0	–	43.7
	Average (58)	32.3	42.4	34.2	31.3	40.0	–	34.7
	Poor or very poor (13)	7.3	3.0	13.2	0.0	20.0	–	7.8
Percent had illness in last month (43)		17.7	40.6	34.2*	25.0	40.0	–	25.9

*P < 0.05. **P = 0.05–0.10. Sum of totals may be less that the total sample (1330) due to missing data

No lower skilled workers were members of the Onyx option

**Table 3:** Utilisation by insurance status

Variable	Uninsured	Privately Insured	GEMS	GEMS OPTIONS			Total sample
				Sapphire/ Beryl/Ruby ‘low-cost’	Emerald ‘mid-cost’	Onyx ‘high-cost’	
Total sample	N = 342	N = 574	N = 408	N = 67	N = 305	N = 36	N = 1330
Mean outpatients visits/person in last month	0.33	0.80	0.74**	0.60	0.74	1.0**	0.66
Percent on chronic medication (385)	15.3	35.0	33.3*	29.2	31.0	52.8*	29.4
Percent any inpatient services in last year (161)	5.9	14.5	14.1*	7.5	15.5	14.3	12.2
Lower skilled workers	N = 97	N = 33	N = 36	N = 16	N = 20	N = 0	N = 168
Mean outpatients visits/person (in last month)	0.44	0.67	0.68*	0.56	0.62	–	0.54
Percent on chronic medication (47)	19.2	42.4	40.5*	43.8	36.8	–	28.7
Percent any inpatient services (in last year) (17)	7.2	21.2	7.9**	0.0	15.0	–	10.1

*P < 0.05. **P = 0.05–0.10

No lower skilled workers were members of the Onyx option

**Table 4:** Type of provider consulted by insurance status of respondent

Provider type	Uninsured	Privately Insured	GEMS	GEMS PACKAGES			Total sample
				Sapphire/ Beryl/Ruby ‘low-cost’	Emerald ‘mid-cost’	Onyx ‘high-cost’	
Total sample	N = 342	N = 574	N = 408	N = 67	N = 305	N = 36	N = 1330
Public Clinic	0.04	0.02	0.02	0.04	0.02	0.06	0.03
Public hospital	0.10	0.03	0.04*	0.03	0.04	0.03	0.05
Private GP	0.10	0.37	0.33*	0.30	0.32	0.44	0.29
Private pharmacy	0.05	0.39	0.29*	0.25	0.28	0.47	0.27
Private Other	0.06	0.21	0.30*	0.10	0.34	0.44	0.20
Lower skilled workers	N = 97	N = 33	N = 36	N = 16	N = 20	N = 0	N = 168
Public Clinic	0.09	0.12	0.08	0.13	0.05	–	0.10
Public hospital	0.16	0.12	0.03	0.00	0.05	–	0.13
Private GP	0.10	0.27	0.36*	0.44	0.30	–	0.20
Private pharmacy	0.06	0.21	0.14	0.06	0.20	–	0.11
Private Other	0.09	0.12	0.2	0.06	0.35	–	0.13

* p < 0.05. Note: No lower skilled workers were members of the Onyx option

**Table 5:** Factors associated with utilization of outpatient and inpatient care among civil servants in South Africa

Variables	Any outpatient services in the last month		Any inpatient admission in past year	
	Univariate OR (95%CI)	Multivariate OR (95%CI)	Univariate OR (95%CI)	Multivariate OR (95%CI)
Age (years)				
20–34	1.0	1.0	1.0	1.0
35–49	1.24 (0.94–1.64)	0.88 (0.63–1.23)	1.32 (0.84–2.06)	1.06 (0.66–1.72)
50–69	1.61 (1.17–2.23)	1.05 (0.70–1.56)	1.41 (0.85–2.35)	1.06 (0.60–1.88)
Gender				
Male	1.0	1.0	1.0	1.0
Female	1.64 (1.31–2.04)	1.52 (1.18–1.95)*	1.96 (1.36–2.81)	1.82 (1.24–2.68)*
Race				
Black	1.0	1.0	1.0	1.0
Coloured	1.42 (1.07–1.88)	1.32 (0.84–2.07)	0.82 (0.52–1.29)	1.26 (1.23–2.68)*
Indian	1.39 (0.87–2.22)	1.22 (0.73–2.06)	1.45 (0.77–2.72)	1.25 (0.63–2.46)
White	1.51 (1.05–2.19)	1.37 (0.87–2.14)	1.06 (0.61–1.84)	1.19 (0.63–2.24)
Marital status				
Married	1.0	1.0	1.0	–
Divorced, widow or separated	1.28 (0.90–1.82)	1.18 (0.80–1.73)	1.35 (0.82–2.21)	
Single	1.01 (0.79–1.29)	1.30 (0.97–1.75)*	0.89 (0.61–1.31)	
Province				
Western Cape	1.0	1.0	1.0	1.0
KwaZulu Natal	0.64 (0.47–0.87)	0.71 (0.45–1.12)	1.45 (0.88–2.37)	1.52 (0.76–3.03)
North West	0.77 (0.57–1.04)	0.73 (0.47–1.14)	1.52 (0.94–2.47)	1.49 (0.76–2.91)
Gauteng	0.74 (0.55–1.00)	0.83 (0.53–1.29)	1.43 (0.88–2.32)	1.49 (0.76–2.92)
Sector				
Health	1.0	1.0	1.0	–
Education	1.49 (1.19–1.87)	1.41 (1.08–1.85)*	1.09 (0.77–1.54)	
Salary grade				
Lower-skilled	1.0	1.0	1.0	1.0
Skilled	0.74 (0.50–1.11)	0.63 (0.40–0.98)*	1.23 (0.66–2.32)	1.15 (0.59–2.27)
Highly skilled	1.21 (0.87–1.70)	0.89 (0.59–1.33)	1.20 (0.69–2.08)	1.07 (0.58–1.99)
Management	1.38 (0.92–2.08)	0.93 (0.57–1.51)	1.53 (0.81–2.89)	1.49 (0.73–3.03)
Overall health status				
Excellent	1.0	1.0	1.0	1.0
Good	1.64 (1.24–2.16)	1.61 (1.20–2.17)*	1.37 (0.84–2.24)	1.30 (0.79–2.16)
Average	2.54 (1.86–3.48)	2.90 (2.04–4.12)*	2.78 (1.69–4.57)*	2.64 (1.55–4.50)*
Poor or very poor	3.73 (1.75–7.92)	4.04 (1.79–9.08)*	5.88 (2.56–13.49)	5.51 (2.27–13.34)*
Insurance status				
GEMS	1.0	1.0	1.0	1.0
Private medical schemes	1.08 (0.84–1.39)	1.00 (0.76–1.32)	1.04 (0.72–1.49)	1.00 (0.68–1.48)
Uninsured	0.35 (0.96–1.42)	0.35 (0.25–0.48)*	0.38 (0.22–0.65)	0.43 (0.25–0.75)*

*p < 0.05 in multivariate analysis; OR odds ratio; Divorced/widow category includes those separated. Multivariate analysis done with logistic regression models

**Table 6:** Factors associated with utilization of publicly and privately provided outpatient care services among civil servants in South Africa

Variable	Any public outpatient services		Any private outpatient visit	
	Univariate. OR (95%CI)	Multivariate OR (95%CI)	Univariate OR (95%CI)	Multivariate OR (95%CI)
Age (years)				
20–34	1.0	1.0	1.0	–
35–49	1.10 (0.60–2.02)	1.63 (0.85–3.14)	1.22 (0.93–1.63)	
50–69	1.76 (0.92–3.36)	1.79 (0.85–3.73)	1.45 (1.05–2.01)	
Gender				
Male	1.0	1.0	1.0	1.0
Female	1.47 (0.92–2.34)	1.40 (0.84–2.33)	1.53 (1.22–1.91)	1.41 (1.08–1.83)*
Race				
Black	1.0	–	1.0	1.0
Coloured	0.75 (0.41–1.37)		1.55 (1.17–2.06)	1.47 (0.93–2.34)
Indian	0.52 (0.16–1.70)		1.60 (1.00–2.56)	1.34 (0.79–2.27)
White	0.61 (0.26–1.44)		1.66 (1.15–2.40)	1.47 (0.94–2.29)*
Marital status				
Married	1.0	–	1.0	1.0
Divorced,/widow	2.08 (1.14–3.80)		1.09 (0.76–1.54)	1.11 (0.75–1.65)
Single	1.24 (0.75–2.05)		1.00 (0.78–1.28)	1.46 (1.09–1.97)**
Province				
Western Cape	1.0	1.0	1.0	1.0
KwaZulu Natal	1.18 (0.62–2.25)	1.26 (0.63–2.53)	0.59 (0.43–0.81)	0.65 (0.40–1.04)*
North West	1.27 (0.68–2.38)	1.44 (0.73–2.85)	0.74 (0.54–1.00)	0.69 (0.44–1.08)
Gauteng	1.22 (0.65–2.28)	1.24 (0.64–2.41)	0.72 (0.53–0.97)	0.84 (0.53–1.32)
Sector				
Health	1.0	1.0	1.0	1.0
Education	0.20 (0.12–0.33)	0.27 (0.16–0.47)*	2.11 (1.67–2.66)	1.88 (1.43–2.48)*
Salary grade				
Lower-skilled	1.0	1.0	1.0	1.0
Skilled	0.50 (0.28–0.88)	0.49 (0.26–0.91)*	1.20 (0.78–1.84)	1.03 (0.63–1.68)
Highly skilled	0.16 (0.09–0.28)	0.37 (0.19–0.69)*	2.37 (1.87–4.46)	1.50 (0.97–2.32)*
Management	0.09 (0.03–0.25)	0.20 (0.07–0.63)*	2.89 (1.87–4.46)	1.69 (1.01–2.81)*
Health status				
Excellent	1.0	1.0	1.0	1.0
Good	0.98 (0.52–1.85)	0.94 (0.48–1.84)	1.76 (1.26–2.22)	1.73 (1.27–2.36)*
Average	2.32 (1.24–4.33)	1.73 (0.87–3.45)	2.21 (1.61–3.04)	2.90 (2.03–4.16)*
Poor or very poor	4.36 (1.57–12.12)	3.11 (0.98–9.88)*	3.05 (1.48–6.29)	4.10 (1.81–9.28)*
Insurance status				
GEMS	1.0	1.0	1.0	1.0
Private scheme	0.58 (0.31–1.08)	0.67 (0.34–1.29)	1.08 (0.84–1.40)	0.96 (0.73–1.26)
Uninsured	2.44 (1.44–4.15)	1.81 (1.02–3.21)*	0.20 (0.14–0.28)	0.21 (0.15–0.31)*

* p < 0.05 in multivariate analysis. Divorced/widow category includes those separated. OR odds ratio. Multivariate analysis done with logistic regression models

## Results

### Socio-demographic characteristics of the study population

Almost two-thirds had tertiary education, and more than half were female (58.7%) (Table 1). Nearly two-thirds were of black African descent, and 10% were white. Almost a third of the respondents were classified as either lower-skilled or skilled, half the respondents were highly-skilled and 15.5% were in management. The median total monthly household income was US$400 in the uninsured, US$667 in those with private insurance and US$546 in GEMS members. Median income varied by GEMS packages, from US$400 in Sapphire/Beryl/Ruby, US$647 in Emerald and US$933 in Onyx.

### Comparing across insurance status and income

#### Insurance and health status

**Fig. 1:**
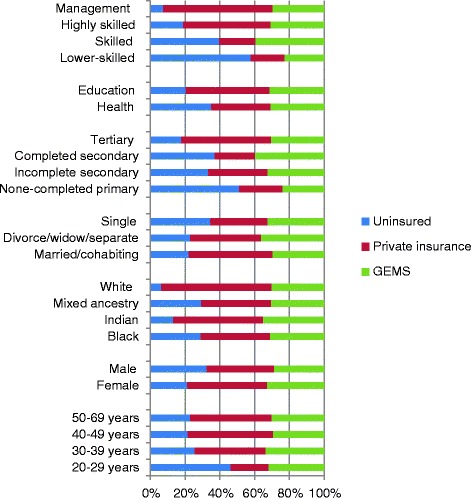
Insurance status, socio-demographic characteristics and salary grade

About a third of respondents were members of GEMS (30.8%), 43.4% were privately insured and a quarter uninsured (25.8%) (Table 1). Membership of GEMS was most common among skilled civil servants, and those who had completed secondary education (Figure 1). Conversely, private insurance was more common among older employees, those with tertiary education, and in management positions. Though overall a quarter were uninsured, these levels were 46.1% in 20–29 year olds, 51.2% in those with primary or no education, and 57.7% in lower-skilled employees. Employees who were single were less likely to have insurance than those married or cohabiting (34.4% versus 22.1%), as were health workers (35.0%), as opposed to those in the education sector (20.4%).

Among the whole study population, the proportion of respondents reporting poor or very poor health status was relatively small across the three insurance categories (2.6% uninsured; 1.7% privately insured; 3.6% GEMS), however nearly twice as many of the insured reported a recent illness than the uninsured (17.3% uninsured; 33.1% privately insured; 32.0% GEMS) (Table 2). A higher proportion of lower-skilled workers reported poor or very poor health (7.8%) than in the overall population (2.6%). These levels were particularly high in lower-skilled GEMS members (7.3% uninsured; 3.0% privately insured; 13.3% GEMS), and again twice as many insured respondents reported a recent illness (17.7% uninsured; 40.6% privately insured; 34.2% GEMS) (Table 2).

### Overall utilisation of any outpatient or inpatient service

#### Insured versus uninsured

The mean number of outpatient visits per month was 0.33 for the uninsured, 0.80 for the privately insured and 0.74 for GEMS members (Table 3). This association was significant in multivariate analysis: the adjusted odds ratio [AOR] of an outpatient visit was 0.35 in the uninsured compared to GEMS members (95%CI = 0.25–0.48; Table 5). The insured were twice as likely to be taking chronic medication in comparison to the uninsured (15% insured; 35.0% privately insured; 33.3% GEMS). Furthermore, nearly three times as many of the insured had been admitted to hospital in the last 12 months compared to the uninsured (5.9% uninsured; 14.5% privately insured; 14.1% GEMS) (Table 3), a finding also noted in multivariate analysis. Similar differentials in utilisation between the uninsured and insured were noted among the lower-skilled group.

### Utilisation by different type of providers and public or private settings

#### Insured versus uninsured among the whole study population

Overall, irrespective of income or insurance status, care was more commonly sought from a private provider than a public provider (Table 4; far right-hand column). For example, the average number of consultations at a private provider was between 0.20–0.29 per month, in comparison to 0.03–0.05 per month at a public provider. The uninsured seldom used public clinics (0.04 visits per month), preferring private GPs and public hospitals for outpatient consultations (each 0.1 mean visits), incurring out-of-pocket payments for both types of visits.^5^ However, utilisation of the private sector providers was higher among the insured than the uninsured. For example, utilisation rates of private GPs were: 0.1 visits for the uninsured, 0.37 for the privately insured and 0.33 visits for GEMS members (Table 4). Multivariate analysis found that similar results with utilisation of private outpatient providers associated with having insurance, higher socio-economic status, as well as poor health status (Table 6, right columns). Analysis, however, showed the opposite was true of utilisation of public outpatient facilities, where adjusted odds of use were highest among the uninsured, and those of lower socio-economic and health status (Table 6; left columns). No major differences were detected between service utilisation in the different provinces.

#### Lower-paid members in comparison to higher paid colleagues

Amongst the lower paid workers, similar to the findings among the whole population, the insured used the private sector general practitioners more than the uninsured, and at a similar level to their better paid colleagues (Table 4). Specifically, the average number of visits to a private GP was 0.36 by lower-skilled GEMS members, and 0.33 visits for GEMS members overall. Multivariate analysis findings of associations between utilisation and pay level are described above.

In sum, in multivariate analysis, GEMS members had higher utilisation of private providers and hospitalisations than their uninsured colleagues. Use of chronic medication was also higher among GEMS than uninsured workers in bivariate analysis. Multivariate analysis also showed that, compared to higher paid workers, the lower paid group were less likely to have an outpatient visit or to be hospitalised, but had higher use of public sector services.

### Comparing effect of the GEMS insurance packages on equity

#### Socio-demographic characteristics, benefit packages and health status

Less than a fifth had chosen the low-cost options (Sapphire, Beryl or Ruby; 16.4%). The majority of GEMS members had selected the mid-cost option (Emerald; 74.8%), and only 8.8% had enrolled in the high-cost option (Onyx) (Table 1). Among the lower-skilled GEMS members, 44.4% had selected low-cost options (more than double the equivalent figure amongst the whole group of civil servants), and just over half (55.6%) had chosen the mid-cost option, and none the high-cost option. Take up of the low-cost options (Sapphire, Beryl or Ruby) was relatively more common among those with either no education or only primary schooling (44.7%), and lower-skilled employees (44.4%). Emerald (the mid-cost option) was more likely to be chosen by those who are Black (80.3%), single (85.1%), and skilled or highly-skilled (75–81%). Onyx (the high-cost option) was chosen predominately by whites (40.0%), those with tertiary education (11.3%), and managers (28.3%; Table 1).

Of the low-cost Sapphire/Beryl/Ruby members, 1.5% reported having a poor or very poor health status, while these figures were 3.6% in mid-cost Emerald and 5.6% in Onyx members (Table 2). The frequency of recent illness was 28.4% in Sapphire/Beryl/Ruby; 31.9% in Emerald and 40% in Onyx members.

### Overall utilisation of any outpatient or inpatient service

#### All GEMS members

Members of the more expensive packages reported a greater number of outpatient visits per month (0.60 visits Sapphire/Beryl/Ruby; 0.74 visits Emerald; 1.0 visits Onyx) (Table 3). About a third of members of the low- and mid-cost packages were taking chronic medication, in comparison to half of the members of the most expensive package (29.2% Sapphire/Beryl/Ruby; 31.0% Emerald; 52.8% Onyx).

#### Lower-paid GEMS members versus higher paid members

Lower-skilled members of the low-cost packages reported 0.56 visits per month, similar to the average visits in the whole population on these packages (0.60 visits), but half that of the overall number of visits for their higher paid colleagues in the high-cost package (1.0 visits) (Table 3). There were no hospital admissions amongst the lower-skilled members of the low-cost package.

### Utilisation by different type of providers, and public or private settings

#### All GEMS members

Members across all packages reported low levels of utilisation of public sector providers, ranging from 0.02–0.06 visits at public clinics, and 0.03–0.04 visits at public hospitals. Though not significant, utilisation of all three private sector services was lowest in cheapest package, intermediate in the mid-cost and most frequent in the high-cost package. Most notably, visits to ‘other’ private providers (dentists, specialists and private hospitals) by Sapphire/Beryl/Ruby members was 0.1, 0.34 in Emerald and 0.44 in Onyx.

#### Lower-paid GEMS members

Lower-skilled members of the low-cost packages reported 0.44 visits to a private GP, the same as the overall levels of Onyx members, but very few visits to a pharmacy (0.06) or ‘other’ provider (0.06).

In sum, overall utilisation was higher among members of the more expensive packages. Yet, the lower-skilled members of the low-cost packages had comparable access to private GPs and chronic medication, although limited access to pharmacies and other providers.

## Discussion

Through GEMS, the South African government has improved access to privately-provided care among government employees, and protected its members from the financial impact of out-of-pocket payments. However, as a whole, the scheme has further institutionalised inequities in access to health care. Firstly, insurance status of government employees and socio-economic status influenced utilisation of care. Moreover, the design of the scheme, based around differential benefit packages, has contributed to inequities in utilisation of services. Members of the higher-cost packages had higher levels of utilisation compared to employees who chose lower-cost insurance packages.

Of particular concern, uninsured government employees continue to incur out-of-pocket costs: they use private GPs only slightly less than the insured government employees, and visit public hospitals more frequently than public clinics, requiring an income-assessed direct payment [17]. Those with lower socio-economic status, the lower-skilled workers, were less likely to be insured, but had four-fold higher levels of poorer health than the overall population. Much of the inequities among government employees noted here would be averted should those who are uninsured join the scheme. Achieving that, however, would involve addressing the reasons for not enrolling, which centre around administrative complexities, lack of information about the scheme, and varying perceptions around the need for health insurance, as noted in other similar studies [22].

Although we did not assess this concern, transport costs may have deterred poorer members from seeking care [23], while supplier-induced demand (common with fee-for-service re-imbursement mechanisms that are used in the South African private sector) may have raised the utilisation levels of better off employees [24]. Interestingly, Onyx members reported higher levels of ill health than the overall population. There are two possible explanations for this: those who could afford to, had chosen higher-cost packages that provided more benefits because they were aware of their ill health, and members of higher-cost packages were more willing to report ill health, because their insurance would cover the costs of care. [25]

More recently, there has been a policy shift towards strengthening the quality of care provided by South Africa’s *public* system [26]. This is due to a recognition by decision-makers that a pre-payment vehicle that requires membership will create a barrier to access (as would happen, for example, by expanding membership of GEMS to the broader population) [24]. By contrast, free access to tax-funded provision does not impose this access barrier [26]. There is an imperative to improve the quality of care for all South Africans, not just the formally employed. This is in line with the South African government’s stated aim to progressively realise ‘*the right to care for* all’ [27]. As quality of public health care hopefully improves, the intention is that government employees (and members of private insurance schemes) will prefer to not pay high insurance premiums, but rather seek care in the public sector [24].

While some steps have been taken to raise the quality of care, little progress has been made with the proposed financing reforms (a centralised pooling of general tax revenue and value-added tax, a purchasing fund, and benefit specification), possibly due to the difficulties of implementing the reforms in a federal system. It therefore remains important to ask whether, despite this shift towards strengthening public sector services, there are advantages to maintaining GEMS from the perspective of the South African government. Firstly, the scheme may have reduced patient burden on the public sector. Secondly, government employees are a key resource in achieving other national objectives, and ensuring their access to care may be a strategic, if inequitable, decision. Lastly, in an already highly inequitable health system, the scheme has facilitated a greater level of cross-subsidisation than otherwise would have taken place, and has enabled lower paid workers’ access to GPs and chronic medication, similar to their better paid colleagues. Nevertheless, future efforts to reduce inequities need to focus on introducing the planned financial reforms in a feasible manner given the capacity available, and be specifically designed so as to meet the needs of the poorer section of society. [28].

The 2010 WHO reports on health care financing contrasts the health system goals of increasing coverage depth (reducing the extent of co-payments), scope (range of benefits covered), and breadth (proportion of the population). [29] Increasing depth or scope for a few will increase inequity, whereas to achieve UHC, a focus on increasing the breadth, or the proportion of the population covered, would ensure more of the population benefit from any rise in the depth and scope of services available. Moreover, fragmented funding pools,^6^ including voluntary health insurance, reduce the possibilities of cross-subsidisation, where the healthy subsidise the sick, and the wealthy the poor [14, 30]. Thus, while GEMS has widened the scope and breadth of coverage for some civil servants, in doing so it has increased inequities in access in the country as a whole [31].

Similar evidence to that presented here has been found in other low- and middle-income countries that have introduced voluntary social health insurance – the relatively affluent benefit more, while the poor gain access to inferior services [32]. A study in Indonesia examining the effects of Askes (a subsidized insurance scheme for government employees) on access to care showed that the insurance scheme had little positive effect on equity in access of care [33]. Similarly, the introduction of health insurance in India was not able to eliminate the inequities in accessing health care services that stem from disparities in socio-economic status [34]. Taking all evidence together, a review of health expenditure data from 138 low- and middle-income countries from the WHO Health Expenditure Database, concludes that voluntary health insurance is not consistently linked to a reduction in out-of-pocket payments, and several countries had exhibited a rise in out-of-pocket payments, as well as a fall in government expenditure in health care [35]. The review concludes “Many countries have paid insufficient attention to the potentially risky role of voluntary health insurance for equitable progress towards UHC. Expanding voluntary health insurance markets bear the risk of increasing fragmentation and inequities.” [35] Going even further, the 2010 World Health Report on health care financing states “it is impossible to achieve universal coverage through insurance schemes when enrolment is voluntary” [36]. Once these schemes are established, they are very difficult to dismantle. In this case, phasing out GEMS would mean that members have to pay premiums for private health insurance without the additional subsidy, or seek care at a public facility. Removing these substantial employee benefit is likely to result in considerable protest from civil servants. The currently beleaguered government is facing almost daily civil society unrest due to failure to deliver a range of services and other concerns around poor governance [37], and is unlikely to take action that would generate protest among its own employees.

The study has several limitations. The cross-sectional design has a limited ability to examine the institutional context within which insurance for civil servants has operated, changes that occurred in the scheme and how these have impacted on equity over time. Also, it is possible that factors influencing enrolment in the long-run vary from those described here in the relatively early stages of the scheme. Further, some study measures were proxies and may incur measurement errors. In particular, use of skill level or salary as a proxy for socio-economic status may be problematic as income can vary over time. Asset indexes, such as type of household dwelling and ownership of a car may be a more valid indicator. We elected to use salary bands as this provides practical information for GEMS management and for similar schemes on which cadre of worker to target. Also, the wide differentials in salary bands suggest that the indicator can adequately differentiate between socio-economic groups. Strengths of the study include sampling of several provinces and two sectors, which raises the generalisability of the findings.

## Conclusion

By establishing the government employees’ medical scheme in 2005, the government at the time chose to prioritise access of civil servants to private sector care, over improving access and quality of public sector services for all South Africans. The scheme has generated inequities due to the differential benefit packages, although, through subsidised membership lower paid civil servants are consulting private GPs and using chronic medication at similar frequencies to their higher paid colleagues. It is unlikely that the scheme will be dismantled or undergo a major reconfiguration, and as a result the inequities have been institutionalised within the health care financing system.

## Abbreviations

GEMS: Government employee medical scheme
LMICs: Low and middle income countries
UHC: Universal health coverage

## Footnotes

1 Horizontal equity refers to ensuring similar access for those with similar need. Vertical equity refers to providing different access to care for those with different need (e.g. better access for those with greater need). In this paper we compare access to care of different members of the scheme on different salaries bands, and therefore with different socio-economic status, and as a result are able to identify where poorer groups have less access to care. This is a situation where horizontal equity hasn’t been achieved, let alone vertical equity.

2 Networks refer to designated health-care providers contracted through the government scheme to provide services to members

3 Although both the public and private sector provide a full range of services to patients, in practice actual provision varies by level of service (clinic, hospital, specialist etc.), as well as the type of provider (clinic, general practitioner, pharmacy, public or private hospital).

4 Retired government employees were not included in the study sample. This population was initially excluded from participation in the government scheme, but later included.

5 Services at public sector primary clinics are free, but a means-assessed fee is charged at public hospitals

6 A country with multiple insurance schemes has fragmented funding pools. In contrast a situation with a single national health service or national health scheme has a single funding pool.
